# Prevalence of Neutralizing Antibodies against Adeno-Associated Virus Serotypes 1, 2, and 9 in Non-Injected Latin American Patients with Heart Failure—ANVIAS Study

**DOI:** 10.3390/ijms24065579

**Published:** 2023-03-14

**Authors:** Julieth A. Sierra-Delgado, Shibi Likhite, Paula K. Bautista, Sergio A. Gómez-Ochoa, Luis E. Echeverría, Elizabeth Guío, Clara Vargas, Norma C. Serrano, Kathrin C. Meyer, Melvin Y. Rincon

**Affiliations:** 1 Centro de Investigaciones, Fundación Cardiovascular de Colombia, Floridablanca 681004, Colombia; 2 Universidad Industrial de Santander, Bucaramanga 680002, Colombia; 3 The Research Institute at Nationwide Children’s Hospital, Columbus, OH 43205, USA; 4 Clínica de Falla Cardíaca y Trasplante, Fundación Cardiovascular de Colombia, Floridablanca 681004, Colombia; 5 College of Medicine, The Ohio State University, Columbus, OH 43205, USA

**Keywords:** neutralizing antibodies, adeno-associated viral vector, heart failure, gene therapy, Colombia

## Abstract

Neutralizing antibody (NAb) activity against the viral capsid of adeno-associated viral (AAV) vectors decreases transduction efficiency, thus limiting transgene expression. Several reports have mentioned a variation in NAb prevalence according to age, AAV serotype, and, most importantly, geographic location. There are currently no reports specifically describing the anti-AAV NAb prevalence in Latin America. Here, we describe the prevalence of NAb against different serotypes of AAV vectors (AAV1, AAV2, and AAV9) in Colombian patients with heart failure (HF) (referred to as cases) and healthy individuals (referred to as controls). The levels of NAb were evaluated in serum samples of 60 subjects from each group using an in vitro inhibitory assay. The neutralizing titer was reported as the first dilution inhibiting ≥50% of the transgene signal, and the samples with neutralizing titers at ≥1:50 dilution were considered positive. The prevalence of NAb in the case and control groups were similar (AAV2: 43% and 45%, respectively; AAV1 33.3% in each group; AAV9: 20% and 23.2%, respectively). The presence of NAb for two or more of the serotypes analyzed was observed in 25% of the studied samples, with the largest amount in the positive samples for AAV1 (55–75%) and AAV9 (93%), suggesting serial exposures, cross-reactivity, or coinfection. Moreover, patients in the HF group exhibited more common combined seropositivity for NAb against AAV1 d AAV9 than those in the control group (91.6% vs. 35.7%, respectively; *p* = 0.003). Finally, exposure to toxins was significantly associated with the presence of NAb in all regression models. These results constitute the first report of the prevalence of NAb against AAV in Latin America, being the first step to implementing therapeutic strategies based on AAV vectors in this population in our region.

## 1. Introduction

Adeno-associated virus (AAV) vectors have emerged as a potential alternative for the delivery of transgenes to somatic tissues in vivo, displaying preferred tissue tropism conferred by its capsids. In addition to the widely known characteristics of AAVs, highlighting their ability to transduce replicative and non-replicative cells, their relatively simple manufacturing process, their minimal risk of insertional oncogenesis [1], and their mild cellular immune response have positioned them as one of the main candidates to serve as vectors for gene therapy [2]. 

However, natural exposure to wild-type AAV serotypes promotes the production of specific antibodies against the AAV capsid, including a sub-class known as neutralizing antibodies (NAb) [3]. High levels of circulating NAb prevent the entry of AAV vectors into the target cell, decrease transduction efficiency and therefore decrease the expression of the therapeutic transgenes. Indeed, the presence of high titers of anti-AAV NAb is one of the main criteria for patient exclusion in clinical trials, and it represents a critical factor in the success of gene therapy based on AAV vectors [4].

Several studies have evaluated the prevalence of NAb against different AAV serotypes, including healthy populations and patients with specific diseases [2,5,6,7]. It has been reported that the levels of anti-AAV NAb can present important variations according to sex, age, and, notably, geographic location [2,8,9]. Furthermore, cross-reactivity (antibodies against one serotype recognizing capsid residues from other serotypes), has also been reported, highlighting the importance of evaluating multiple serotypes in any population [8,9]. Despite its critical role and the important geographical variations in its prevalence, so far, only one study has evaluated the prevalence of anti-AAV total antibodies (AAV2, AAV5, AAV6, AAV8, and AAVrh10) in a Latin American population, highlighting the lack of information on specifically NAb in the subcontinent [10]. There is currently no data on the prevalence of NAb against any AAV serotypes in Latin America in healthy patients [11]. Thus, the assessment of the prevalence of NAb against AAV vectors plays a fundamental role as a starting point for AAV-based clinical trials, especially for high-impact pathologies in Latin American health systems, such as cardiovascular disease (CVD). 

CVD, particularly heart failure (HF), represents a significant public health problem due to its high prevalence, poor prognosis, and high healthcare costs [12]. Approximately 1–2% of adults in developed countries suffer from HF, with a prevalence of ≥10% among people 70 years or older [13]. It is estimated that in Latin America, HF and its derived complications are responsible for 6.3% of total deaths every year [12,13], with a mortality rate of 4.46 deaths per 100,000 inhabitants in countries such as Colombia [14]. Pharmacological and non-pharmacological therapies provide notable clinical improvement and reduce the progression of HF; nonetheless, these approaches have not been enough to mitigate the burden of this disease [12]. In this context, the significant improvement in the current understanding of pathophysiological and molecular mechanisms involved in HF has favored the design and initiation of gene therapy trials in this area [15]. Despite discouraging initial results, more recent studies evaluating more efficient viral vectors and promising new potential molecular targets keep open the prospects for improving the evolution and survival of HF patients [15].

Finally, one of the most relevant considerations in this context corresponds to the evidence of seropositivity for different AAV serotypes in the same individual, which makes it essential not only to establish the prevalence of NAb against a specific serotype but also to assess the combined prevalence of the main serotypes used in AAV-based therapy for HF. In this study, we evaluated the prevalence of anti-AAV NAbs against two serotypes commonly used for cardiac gene therapy, namely AAV1 and AAV9, due to their defined cardiac tropism, together with the most prevalent serotype worldwide, AAV2, in a sample of patients with HF and healthy donors from Colombia [2]. 

## 2. Results

**Figure 1:**
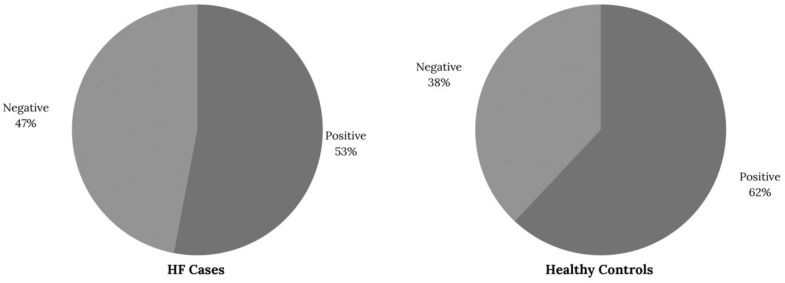
The proportion of patients with positive results for the presence of NAb against at least one of the three serotypes evaluated according to the group.

The salient demographic features of the cohort, which list the sociodemographic and clinical characteristics, are presented in Table 1. Samples of 60 cases with HF and 60 healthy controls were examined. Patients in the HF group lived more frequently in rural areas and reported a higher prevalence of hypertension, dyslipidemia, renal disease, and acute myocardial infarction history. Furthermore, most patients in the HF group were in stage B according to the ACC/AHA classification (Table 1). In total, 53.3% (*n* = 32) of the HF cases and 63.3% (*n* = 38) of the participants in the healthy donor group were positive for at least one of the three serotypes evaluated (Figure 1). 

### 2.1. NAbs Prevalence and Differences between the Analyzed Groups

**Figure 2:**
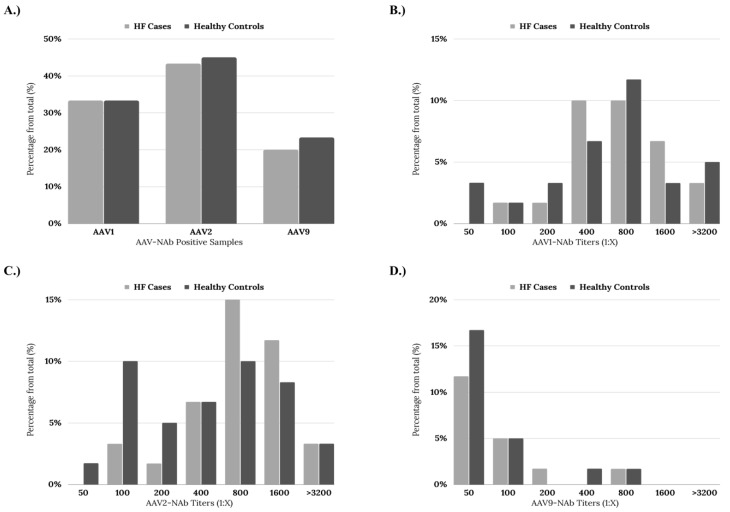
(**A**) Bar graph summarizing the prevalence of Nab against each AAVs according to the group. Bar graph of the prevalence of NAb against each AAVs according to the titer measured (**B**) anti-AAV1 NAb titers. (**C**) anti-AAV2 NAb titers. (**D**) anti-AAV9 NAb titers.

When the seroprevalence of NAbs against each individual AAV was evaluated, it was found that 33.3% of the samples (*n* = 20) were positive for AAV1 in both groups (Figure 2A). Additionally, 43.3% of the HF cases (*n* = 26) and 45% of the healthy donors (*n* = 27) were positive for AAV2 (Figure 2A). Finally, anti-AAV9 NAbs were positive in 20% of the HF cases (*n* = 12) and 23.33% of the healthy donor group (*n* = 14) (Figure 2A). Overall, there was no statistically significant difference in the prevalence of individual NAbs between the groups across the different evaluated titers (Figure 2B–D). 

### 2.2. The Combined Prevalence of NAbs Anti-AAV in HF Patients

Regarding the combined prevalence among the three serotypes evaluated, 24.9% of the participants in the HF group presented NAb against two or more serotypes, while, in the healthy donor group, the prevalence for two or more serotypes was estimated to be 31.7%. There were no significant differences between the groups (*p* = 0.68). Interestingly, in contrast to the HF cases, participants in the control group presented a different distribution of combined prevalence, with a lower proportion of positive samples for the three serotypes (6.7% vs. 18.3% in the HF group); however, this difference was not statistically significant (*p* = 0.053) (Table 2). Nevertheless, patients in the HF group exhibited more common combined seropositivity for NAb against AAV1 and AAV9 than those in the control group (91.6% vs. 35.7%, respectively. *p* = 0.003. Table 2).

### 2.3. Socioeconomic Factors Related to the Prevalence of NAbs Anti-AAV

To evaluate the role of sociodemographic factors in the prevalence of anti-AAV NAb, we assessed the association between age and sex with the presence of NAb in each group, since these are two of the main variables associated with the presence of Nab against AAV in the literature [5]. In the HF cases, there were no statistically significant differences between the presence of NAb by sex (*p* = 0.71) or age (*p* = 0.37). Similarly, there was no association between age or sex and NAb presence in the control group (age *p* = 0.37, sex *p* = 0.74). To evaluate the impact of other factors and to determine possible interactions between the independent variables, binomial logistic regression models were fitted with the presence of NAb for at least one of the serotypes as the dependent variable. The final model had an area under the ROC curve of 0.849 and included the following factors: toxic exposure, previous surgery, access to drinking water, access to garbage collection, previous hospitalization, and travel in the last 12 months. Of these variables, toxic exposure and a previous surgery showed a statistically significant association (Table 3).

We then evaluated the impact of socioeconomic factors on the prevalence of NAb for more than one serotype using multinomial logistic regression. The presence of NAb was used as the output variable and was divided into three categories: “NONE” for the samples that did not present NAb, “ONE” for the samples that presented NAb against only one serotype, and "MULTIPLE" for the samples that presented NAb against two or more serotypes. The NONE category was chosen as a reference. The final model included the following variables: toxins exposure, allergies, previous hospitalization, access to sewage, place of residence, and access to drinking water. Even though the model was statistically significant, the quality of fit was suboptimal per McFadden’s (0.140) and Cox-Snell´s (0.262) pseudo-R^2^ statistics. No variable was statistically significant in the “MULTIPLE” category, while in the “ONE” category, the significant variables were toxic exposure and previous hospitalization, similar to the results of the binomial logistic regression (Table 4).

## 3. Discussion

In this cross-sectional study, the most prevalent anti-AAV NAb in this Colombian cohort was against the AAV2 serotype, followed by AAV1 and AAV9. In addition, the prevalence of anti-AAV NAb against one serotype was similar between groups (HF patients vs. healthy controls); however, there were different patterns of seropositivity for more than one NAb, with patients in the HF group showing a higher prevalence of NAbs against AAV1 and AAV9 concomitantly present. Finally, we identified that some conditions were significantly associated with the presence of at least one of the three anti-AAV NAb in this population, such as toxic exposure and previous hospitalization/surgery. This work constitutes the first study to report the specific prevalence of NAbs against AAV in Colombia and Latin America.

Since 1968, the year in which the first study evaluating the prevalence of antibodies against AAV was carried out, multiple studies have estimated the seroprevalence of these NAbs around the world in several populations, with those against AAV1, 2, 3, 4, 5, 6, 7, 8, 9, and 10 being mainly investigated because these are currently the most frequently studied viruses as candidates for gene therapy (Supplementary Table S1) [16,17]. However, the natural limitations of these viruses have encouraged the development of newly engineered AAV candidate vectors, which, through high-throughput molecular engineering approaches, present superior characteristics to their natural counterparts, highlighting a greater specificity to transduce specific cell lineages and neutralize antibody-evasion capabilities, among others [18]. Unfortunately, despite the advances made in the immune evasion capabilities of these engineered vectors, it has been observed that many of the modifications in their capsid structure may compromise their functionality [19,20]. In addition, the presence of cross-reactivity by NAb with the modified capsid structures has been reported, resulting in the production of new antibodies against these structures [18]. Therefore, the evaluation of the presence of these anti-AAV neutralizing antibodies continues to be of great importance for the assessment of the efficacy of this vector approach in gene therapy.

Regarding current data, population-based studies conducted at a global level have reported important differences in the prevalence of NAb according to the serotype evaluated, the population studied, and the NAb detection method used, the latter being a critical factor when comparing the results of the different studies [9,10,21,22]. This is due to the fact that AAV antibody assays are not currently standardized globally, so the cutoff points for defining positivity in each study are assay-specific [10,23]. Among the types of assays currently available are neutralizing antibody assays and total antibody assays, the latter being mainly ELISA-based assays with the ability to detect both neutralizing and non-neutralizing antibodies. Despite their convenience, reproducibility, and sensitivity, their positivity in the presence of non-neutralizing antibodies excludes a large proportion of potential candidates for gene therapy [23].

In addition to the prevalence data, the analysis of factors associated with the presence of these antibodies acquires a key value in identifying those populations whose associated characteristics would make them suboptimal candidates for gene therapy with these viral vectors. Among these factors, the increase in the prevalence of NAb with increasing age is a factor currently debated in the literature, and there are a considerable number of studies whose results have supported this hypothesis [9,10,21,24,25]. For example, the study by Klamroth et al., in which 546 patients with hemophilia A in nine countries were evaluated, reported significantly higher seropositivity rates in adults compared to adolescents and in older adults compared to young adults. This result may be due to the fact that AAVs are endemic viruses, so a higher exposure in older individuals is to be expected [10]. However, as is the case in the present investigation, some studies have not reported significant variations with age [6,22,26]. This could be due to the inclusion of mainly adult individuals in these studies, as previous evidence suggests that seroconversion may occur mainly during early childhood for most serotypes [27]. Furthermore, other studies suggest that antibody titers tend to remain stable over time in adults and that the rate of seroconversion in this population is very low and has minimal geographical variations [10,28]. However, at present no study has established a clear stratification of the prevalence of these antibodies by age group. 

On the other hand, we did not find a relationship between the presence of NAb for all serotypes and sex in our sample. A potential relationship between sex and NAb presence is controversial and is still subject to debate in the field; however, most studies have not found a significant association between these two variables [2,6,21,22,29]. Among other known demographic variables, Ling et al. previously reported a significant correlation between the presence of NAb and the nutritional status of the participants characterized according to the categories of traditional Chinese medicine [29]. However, the present study did not observe any association between the body mass index, presence of obesity/overweight, and any lifestyle factor (exercise, smoking, alcohol) with the prevalence of NAb in the participants in either group.

Interestingly, we observed a significant association between toxic exposure (air pollutants, heavy metals, and pesticides) and NAb presence in both fitted models. This association could be explained by environmental factors or by the interaction of toxic nanoparticles with the immune system at the time of exposure to AAV [30]. However, further studies with a larger number of participants are needed to elucidate the real role of toxicological exposure in the prevalence of anti-AAV NAb. Another variable that was significantly associated with the presence of NAb was previous hospitalization/surgeries, the association of which can potentially be explained by higher exposure to these viruses in the hospital setting. We should highlight that the multinomial logistic model, despite producing significant results for the aforementioned variables, had a regular quality of fit according to the McFadden (0.140) and Cox-Snell (0.262) pseudo-R2 statistics. Therefore, future studies should evaluate more population variables, with a larger sample size to allow for a better quality of fit in the statistical models used, thus allowing to elucidate more adequately the variables associated with seropositivity for these antibodies.

### Study Strengths and Limitations

The population selected for the study presented a similar trend in their demographic variables, including age and sex (Table 1), which increases the validity of the matching process and the statistical analysis performed. On the other hand, one of the limitations of the study is the geographic location of the participants in the healthy donor group. Although the HF cases had a wide geographic variation in origin, more healthy donors were living in urban areas of a specific region than participants in the HF group. This limitation restricts the extrapolation of the results to other areas of the country and causes a loss of statistical power, especially when analyzing socioeconomic variables and access to public services. For this reason, a matching of residence (in an urban or rural area) in addition to sex and age in future studies should be considered. However, the present study provided insights into the prevalence and factors associated with anti-AAV NAb in Latin American patients with HF and healthy individuals.

## 4. Materials and Methods

### 4.1. Patients and Samples

Serum samples were collected from 60 participants of the outpatient Heart Failure Clinic at Fundación Cardiovascular de Colombia (FCV) and 60 healthy donors, with inclusion and exclusion criteria as previously described (13). Healthy donors were included by active search in the community, matched according to age and sex with the HF cases group. The Colombian Administrative Department of Science, Technology, and Innovation (COLCIENCIAS) and the ethics board of the FCV approved the study. This research adhered to the tenets of the Declaration of Helsinki. All participant subjects signed an informed consent previous participation in the study.

Each participant underwent peripheral blood sampling through antecubital venous puncture with the Vacutainer^®^ system, using two 5 mL yellow cap tubes. The tubes were centrifuged for 10 min at 3200 revolutions per minute (rpm) to perform the fractionation of components. The serum was transferred in cryovials of 1 mL and stored at −80 °C until processing. Serum samples were thawed on wet ice, and no sample was subjected to freeze-thaw cycles. Additionally, a survey was conducted for each participant to determine epidemiological variables and socioeconomic conditions.

### 4.2. Cell Culture and Neutralizing Antibody Assay

The human embryonic kidney cell line, HEK293, was maintained in Dulbecco’s modified Eagle’s medium, high glucose (DMEM; Sigma Aldrich, St. Louis, MO, USA) supplemented with 10% fetal bovine serum (FBS; Sigma Aldrich) and 1% Penicillin/Streptomycin (P/S; Sigma Aldrich) until they reached 80% confluence. AAV vectors were acquired commercially from ViGene Biosciences (Rockville, MD, USA) and SAB Tech Inc (Philadelphia, PA, USA).

The in vitro neutralizing antibody assay protocol was developed by the Meyer lab and is a modified version of a previously published protocol [11,31]. As a brief introduction to the topic, in the setting of Nab detection, the option of in vivo or in vitro assays is available. We selected in vitro assay, as it is considered the gold standard due to its higher correlation with in vivo transduction and its lower cost compared to an in vivo test. First, a 96-well culture plate was pre-treated for 30 min with fibronectin (FC010, Merck) at a density of 2 ug/cm^2^. Then, 2 × 10^4^ HEK293 were seeded into the 96-well culture plates in 100 mL of DMEM supplemented with 10% FBS and 1% of Penicillin/Streptomycin and let rest for 24 h. On the day of transduction, serum samples from HF cases or healthy donors serially diluted 2-fold in DMEM (starting dilution of 1:10) were incubated with an AAV vectors expressing the green fluorescent protein (GFP) under the control of the CMV enhancer/beta-actin (CB) promoter (rAAV-CB-GFP) (AAV1, AAV2: ViGene Biosciences, Rockville, MD, USA; AAV9: SAB Tech Inc, Philadelphia, PA, USA) in the same medium containing a specific number of vector genome copies per cell (MOI): (AAV1:12500; AAV2: 5000, AAV9: 25000) in a 1:1 volume ratio for 1 h at 37 °C. Following the incubation, the entire mixture of diluted serum and AAV vector was added to the cultured cells that were washed three times with DMEM with 1% of P/S before the serum/AAV treatment. The vector doses were pre-determined to obtain a semi-saturated optical density (OD) value for each serotype. This measure was selected due to the need of obtaining linear readings for the GFP and the observation of an association between a higher multiplicity of infection and the saturation of GFP (Supplementary Figure S1). For rAAV9 transfection, additional cell treatment with neuraminidase III (N7885, Sigma Aldrich) at 5 mEq/well for 2 h was necessary to allow viral uptake. After 24-h incubation time, GFP expression was quantified using a plate reader (Synergy 2, Biotek, Winooski, VT, USA) and a laser-based confocal imaging platform (InCell 6000, GE Healthcare Life Sciences, Chicago, IL, USA). The neutralizing titer of the sample was reported as the first dilution at which ≥50% inhibition of the GFP signal compared to the negative control was achieved. A neutralizing titer of greater than 1:50 was considered positive, being the cutoff determined considering the wide range of titer values reported in the literature (1:2 to 1:100).

In order to achieve a semi-saturated optical density (OD), we sought to obtain linearity in our readings for green fluorescent protein (GFP). We found that higher multiplicities of infection (MOIs) resulted in a saturation of relative fluorescence units (RFUs) and, consequently, a 50% reduction in virus did not result in a 50% reduction in GFP signal. Please refer to the Supplementary Figure S1 I have provided for further clarification. Additionally, Meliani’s paper states that "In general, the optimal MOI to be used in the NAb assay should correspond to the lowest amount of virus resulting in a reporter gene signal above background and not saturated". 

### 4.3. Statistical Analysis

The chi-square test and logistic binomial and multinomial regressions were used for the statistical analysis of the results. A *p*-value < 0.05 was considered statistically significant. The variables for the logistic regression models were chosen using the forward stepwise method. Model selection was made using the Akaike information criterion (AIC). To evaluate the overall fit of the binomial model, the Hosmer-Lemeshow goodness of fit test was performed. For the multinomial logistic regression, the statistical validation of the model was performed using a chi-square distribution and a chi-square test of the likelihood ratio with a saturated model. The quality of fit of the logistic regression models was validated using McFadden’s and Cox-Snell’s’ pseudo-R2 statistics. All statistics were performed using the software R, version 3.3.3. 

## 5. Conclusions

In conclusion, the prevalence of NAb in the Colombian population is significant, with more than half of the participants having antibodies against at least one AAV, and NAb against AAV1 and AAV2 being observed in one out of every three individuals. However, most of the patients evaluated could be eligible for different gene therapy clinical trials currently being developed, postulating Colombia as a favorable site for carrying out future therapeutic trials for different conditions. Additionally, a potential association between exposure to toxins and seropositivity against these viruses was evidenced, a finding that opens the door to a generation of hypotheses about the possible mechanisms underlying this association. These results constitute the first report of the prevalence of anti-AAV NAb in HF in Latin America, being the first step to implementing therapeutic strategies based on AAV vectors in this population in our region.

## Figures and Tables

**Table 1:** Main sociodemographic characteristics of the sample.

	HF Cases (N = 60)	Healthy Controls (N = 60)	*p*-Value
Age	62 (51.8, 72)	61 (52, 71)	0.655
Male sex	46 (76.7%)	46 (76.7%)	1.000
Race			0.074
Hispanic	55 (91.7%)	60 (100.0%)	
African-American	4 (6.7%)	0 (0.0%)	
White	1 (1.7%)	0 (0.0%)	
Origin			**0.032**
Rural Area	36 (57,9%)	30(50%)	
Urban Area	24 (42,1%)	30(50%)	
T2DM	12 (20.0%)	9 (15.0%)	0.471
Hypertension	35 (58.3%)	24 (40.0%)	**0.045**
Dyslipidemia	21 (35.0%)	8 (13.3%)	**0.015**
Renal Disease	16 (26.7%)	5 (8.3%)	**0.008**
Obesity/Overweight	29 (48.3%)	37 (61.7%)	0.142
AMI	26 (43.3%)	2 (3.3%)	**<0.001**
Years with HF	4 (2, 7)		NA
HF Stage			NA
A	1 (1.7%)	-	
B	49 (81.7%)	-	
C	10 (16.7%)	-	
Hospitalized during the last year	40 (66.7%)	0 (0%)	NA
Physical activity	43 (71.7%)	42 (70.0%)	0.841
Minutes of exercise per day	30 (0, 60)	30 (0, 60)	0.603
Exercise days per week	5 (0, 7)	2 (0, 6)	**0.047**
Surgery during the last year	46 (76.7%)	38 (63.3%)	0.111
Toxic exposure during the last year	16 (26.7%)	21 (35.0%)	0.323
Allergies	11 (18.3%)	8 (13.3%)	0.453
Hemoglobin	13.3 (12.2, 14.2)	-	NA
Leukocytes	6.9 (5.6, 8.2)	-	NA
Platelets	213 (178.5, 250.5)	-	NA
LVEF	30 (20, 45)	-	NA
Garbage disposal	52 (86.7%)	60 (100.0%)	**0.003**
Sewage system	51 (85.0%)	60 (100.0%)	**0.002**
Traveling during the last year	29 (48.3%)	8 (13.3%)	**<0.001**
AAV1 Seropositivity	20 (33.3%)	20 (33.3%)	1.000
AAV2 Seropositivity	26 (43.3%)	27 (45.0%)	0.854
AAV9 Seropositivity	12 (20.0%)	14 (23.3%)	0.658

Abbreviations: T2DM: Type 2 Diabetes Mellitus; AMI: Acute Myocardial Infarction; HF: Heart Failure; LVEF: Left Ventricle Ejection Fraction. Variables without information in a group are labeled with a hyphen.

**Table 2:** Combined prevalence of NAb against AAV1, AAV2, and AAV9 in HF cases and healthy donors.

	AAV1 ^†^	AAV2 ^†^	AAV9 ^†^
HF cases			
AAV1 ^†^	-	75% AAV2 ^†^ (*n* = 15/20)	55% AAV9 ^†^ (*n* = 11/20)
AAV2 ^†^	57.6% AAV1^+^ (*n* = 15/26)	-	42.3% AAV9 ^†^ (*n* = 11/26)
AAV9 ^†^	91.6% AAV1^+^ (*n* = 11/12) ^†^	91.6% AAV2 ^†^ (*n* = 11/12)	-
Healthy donors			
AAV1 ^†^	-	50% AAV2 ^†^ (*n* = 10/20)	25% AAV9 ^†^ (*n* = 5/20)
AAV2 ^†^	37% AAV1 ^†^ (*n* = 10/27)	-	44.4% AAV9 ^†^ (*n* = 12/27)
AAV9 ^†^	35.7% AAV1 ^†^ (*n* = 5/14)	85.8% AAV2 ^†^ (*n* = 12/14)	-

^†^ Significantly higher prevalence in HF cases than in healthy donors (*p* = 0.003). Comparisons between the same group are labeled with a hyphen.

**Table 3:** Variables included in the binomial logistic regression model.

	OR	CI (95%)	*p*-Value
Toxic exposition	22.72	[20.42; 25.01]	0.007
Previous surgery	14.65	[12.59; 16.74]	0.015
Access to potable water	6.00 × 10^−10^	[3.4 × 10^−156^; 4744]	0.999
Access to proper garbage disposal	3.3010^−9^	[NA; 3.83 × 10^113^]	0.999
Previous hospitalization	4.44	[2.89; 5.98]	0.058
Travel in the last year	0.29	[0.85; 15]	0.093

OR: Odds Ratio, CI: Confidence Interval, NA: Not available.

**Table 4:** Variables included in the multinomial logistic regression model.

CATEGORY	Independent Variable	OR	CI (95%)	*p*-Value
“ONE”: Positive for only one serotype evaluated	Toxic exposition	2.97	[1.03; 8.56]	0.001
	Allergies	3.05	[0.8; 11.3]	0.43
	Previous hospitalization	3.58	[0.8; 15.2]	0.001
	Sewerage system	9.48 × 10^−10^	[6.6 × 10^−122^; 1.35 × 10^103^]	0.24
	Place of residence	4.01 × 10^−20^	[5.1 × 10^−33^; 3.15 × 10^−7^]	0.95
	Access to potable water	3.33 × 10^−11^	[1.78 × 10^−110^; 6.22 × 10^88^]	0.43
“MULTIPLE”: Positive for multiple serotypes evaluated	Toxic exposition	0.55	[0.1; 1.54]	0.41
	Allergies	0.87	[0.2; 3]	0.41
	Previous hospitalization	0.5	[0.07; 3.3]	0.12
	Sewerage system	2.93 × 10^−15^	[6.27 × 10^−179^; 1.37 × 10^149^]	0.47
	Place of residence	7.53 × 10^−21^	[9.57 × 10^−34^; 5.9 × 10^−8^)	0.97
	Access to potable water	1.88 × 10^−5^	[5.2 × 10^−182^; 6.7 × 10^171^]	0.95

OR: Odds Ratio, CI: Confidence Interval.

## Data Availability

The data used in this manuscript will be made available from the corresponding authors upon reasonable request.

## Supplementary Materials

The following supporting information can be downloaded at: https://www.mdpi.com/article/10.3390/ijms24065579/s1. References [2,5,6,8,9,10,16,17,21,22,24,25,27,29,31,32,33,34,35,36,37,38,39,40,41,42,43] are cited in the Supplementary Materials File.
