# Supplementary material for: Prevalence of Neutralizing Antibodies against Adeno-Associated Virus Serotypes 1, 2, and 9 in Non-Injected Latin American Patients with Heart Failure—ANVIAS Study
Source: Int J Mol Sci. 2023 Mar 14;24(6):5579. doi: 10.3390/ijms24065579 (PMC10051173; doi:10.3390/ijms24065579)
Supplement: Supplementary file 1 [file ijms-24-05579-s001.zip › ijms-2128077-supplementary.pdf]

Prevalence of Neutralizing Antibodies against Adeno-Associated Virus Serotypes 1, 2, and 9 in Non-Injected Latin American Patients with Heart Failure—ANVIAS Study

Supplementary File

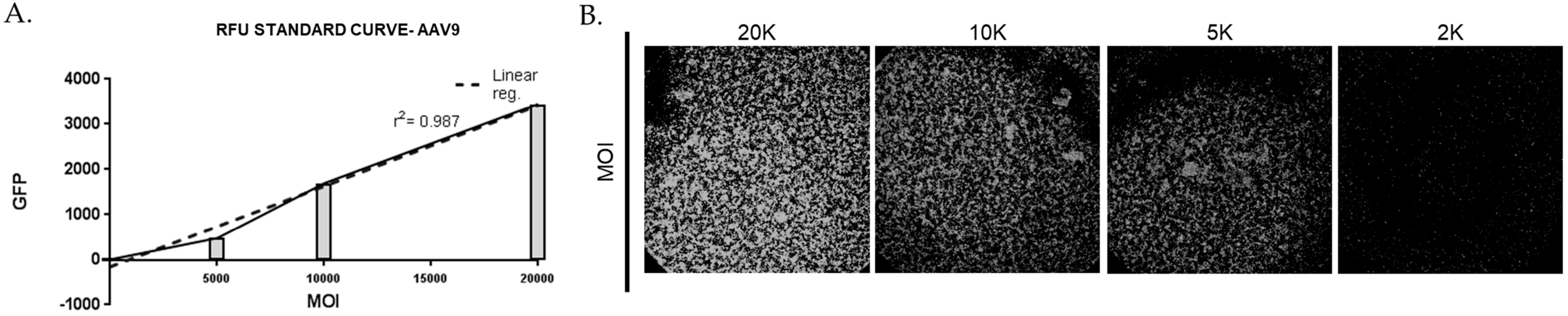

Supplementary Figure S1. (**A**) Image of a calibration curve using RFU (OD). Samples that showed a 50% of reduction in signal according to the standard were considered positive. (**B**) Representative images from the automated high throughput microscopy system (Incell 6000, GE, USA) showing qualitative linear diminution in signal correlated with the MOIs.

Supplementary Table S1. Non-systematic compilation of studies that have evaluated the prevalence of neutralizing antibodies to various serotypes of adeno-associated viruses in different populations.

Method	Country	Year	Age group	Titer cut-off	AAV1	AAV2	AAV3	AAV4	AAV5	AAV6	AAV7	AAV8	AAV9	BAAV	Target population	Ref.
Nab in vitro	USA	1968	18-30 years	1:10	20	40	30	0	–	–	–	–	–	–	Healthy	[16]
			5-10 years		40	60	60	0	–	–	–	–	–	–		
Nab in vitro	USA	1968	3 months-3 years	1:10	–	36.6	40.5	–	–	–	–	–	–	–	Healthy	[17]
ELISA/Nab in vitro	USA	1999	18-54 years	1:20	–	32	–	–	–	–	–	–	–	–	Healthy	[32]
			18-52 years		–	32	–	–	–	–	–	–	–	–	Cystic fibrosis	
ELISA	Germany	1999	20-29 years	NR	–	40	–	–	–	–	–	–	–	–	Healthy	[31]
	Brazil				–	60	–	–	–	–	–	–	–	–		
	Japan				–	40	–	–	–	–	–	–	–	–		
Nab in vitro	USA	2002	16-44 years	1:20	–	22	–	–	–	–	–	–	–	–	Cystic fibrosis	[33]
Nab in vitro	EE.UU.	2006	18-47 years	1:20	–	30	–	–	14	30	–	–	–	–	Healthy	[5]
			0-17 years		–	12.4	–	–	6.7	9	–	–	–	–	Cystic fibrosis	
Nab in vitro	USA	2009	NR	1:20	20	28	–	–	–	–	12	14	–	–	NR	[2]
	Australia				30	35	–	–	–	–	29	27	–	–		
	Europe				27	35	–	–	–	–	25	22	–	–		
	Africa				43	56	–	–	–	–	31	31	–	–		
ELISA/Nab in vitro	USA	2009	18-77 years	1:3,1	–	37.5	–	–	–	–	–	–	–	–	Healthy	[34]
ELISA/Nab in vitro	France	2010	25-64 years	1:20	50.5	59	–	–	3.2	37	–	19	33.5	–	Healthy	[35]
Nab in vitro	USA	2011	0-18 years	1:20	–	22	–	–	–	–	–	15	–	–	Healthy	[8]
Nab in vitro	Netherlands	2011	20-69 years	1:100	79	50	–	31	77	67	–	94	–	–	Healthy	[36]
			16-84 years		63	50	–	44	53	85	–	70	–	–	IBD	
Nab in vitro	USA	2012	9 months-7 years	1:2	–	43.6	–	–	25.8	–	–	22.5	–	–	Hemophilia	[27]
ELISA/Nab in vitro	France	2012	NR	1:2	59	–	–	–	–	–	–	–	–	–	Healthy	[37]

Nab in vitro	China	2013	<18-56 years	1:10	69.8	–	–	–	–	–		–	–	–	Healthy	[43]
Nab in vitro	China	2013	<18-56 years	1:10	–	96.6	–	–	40.2	–	–	82	–	–	Healthy	[43]
			5-64 years		–	–	–	–	37	–	–	–	–	–	HIV +	
Nab in vitro	EE. UU.	2013	17-72 years	1:25	–	17	–	4	4	–	–	–	–	15	Healthy	[38]
			24-76 years		–	24	–	8	15	–	–	–	–	24	Sjögren syndrome	
Nab in vitro	Japan	2014	12->52 years	1:14	36.5	35.3	–	–	37.6	–	–	32.9	32.9	–	Healthy	[39]
					39.7	28.8	–	–	35.6	–	–	32.9	27.4	–	Hemofilia	
Nab in vitro	Italy	2015	5-25 years	1:5	–	–	–	–	–	–	–	14	–	–	MPS IV	[40]
	Netherlands				–	–	–	–	–	–	–	27	–	–		
	Türkiye				–	–	–	–	–	–	–	67	–	–		
Nab in vitro	China	2015	18-< 51 years	1:20	–	92	89	–	–	–	–	69	–	–	Healthy	[29]
Nab in vitro	Belgium	2016	18-80 years	1:02	62	–	–	–	–	–	–	–	–	–	Heart Failure	[9]
	Sweden				48	–	–	–	–	–	–	–	–	–		
	Netherlands				73	–	–	–	–	–	–	–	–	–		
	United Kingdom				65	–	–	–	–	–	–	–	–	–		
	Germany				64	–	–	–	–	–	–	–	–	–		
	Hungary				79	–	–	–	–	–	–	–	–	–		
	Poland				79	–	–	–	–	–	–	–	–	–		
	Israel				75	–	–	–	–	–	–	–	–	–		
	Denmark				59	–	–	–	–	–	–	–	–	–		
	USA				32-67	–	–	–	–	–	–	–	–	–		
Nab in vitro	USA	2016	2-31 years	1:20	–	20	–	–	–	–	–	22	24	–	Methylmalonic acidemia	[41]
Nab in vitro	USA	2018	NR	1:16	–	–	–	–	–	–	–	–	17	–	Healthy	[42]
Nab in vitro	Europe	2019	5-26 years	1:1	–	–	–	–	–	–	–	31	–	–	Crigler-Najjar Syndrome	[24]
ELISA/Nab in vitro	USA	2019	19-84 years	1:5	–	48	–	–	–	–	–	41	–	–	Hemophilia	[6]
	USA				–	56	–	–	22	–	–	32	–	–	Healthy	
	Europe				–	53	–	–	32	–	–	60	–	–	Healthy	
Nab in vitro	India	2020	5 years-NR	1:5	–	–	91	–	–	–	–	–	–	–	Hemophilia	[25]
			>18 years		–	–	65	–	–	–	–	–	–	–	Healthy	

Nab in vitro	France	2022	36.0 ± 14.9	NR	–	61	–	–	27	55	–	45	–	–	Hemophilia	[10]
	Germany		36.0 ± 14.9		–	48	–	–	37	44	–	43	–	–		
	Italy		36.0 ± 14.9		–	45	–	–	28	40	–	40	–	–		
	Japan		36.0 ± 14.9		–	43	–	–	30	31	–	39	–	–		
	Russia		36.0 ± 14.9		–	64	–	–	46	56	–	54	–	–		
	South Africa		36.0 ± 14.9		–	95	–	–	52	80	–	70	–	–		
	UK		36.0 ± 14.9		–	65	–	–	6	41	–	41	–	–		
	USA		36.0 ± 14.9		–	54	–	–	27	38	–	31	–	–		
Nab in vitro	Japan	2022	10-70 years	1:1	22	25	–	–	24	23	24	20	21	–	Hemophilia	[21]
			10-70 years		22	27	–	–	23	21	22	22	29	–	Healthy	
ELISA/Nab in vitro	USA	2022	20-80 years	1:5	37	–	–	–	29	–	–	–	–	–	Healthy	[22]

Variables without information in a group are labeled with a hyphen.
